# Charge Localization in Acene Crystals from *Ab Initio* Electronic Structure

**DOI:** 10.1021/acs.jpclett.3c00191

**Published:** 2023-03-30

**Authors:** Francesco Ambrosio, Julia Wiktor, Alessandro Landi, Andrea Peluso

**Affiliations:** † Dipartimento di Chimica e Biologia Adolfo Zambelli, Università di Salerno, Via Giovanni Paolo II, I-84084 Fisciano (SA), Italy; ‡ Dipartimento di Scienze, Università degli Studi della Basilicata, Viale dell’Ateneo Lucano, 10-85100 Potenza, Italy; ¶ Department of Physics, Chalmers University of Technology, SE-412 96 Gothenburg, Sweden

## Abstract

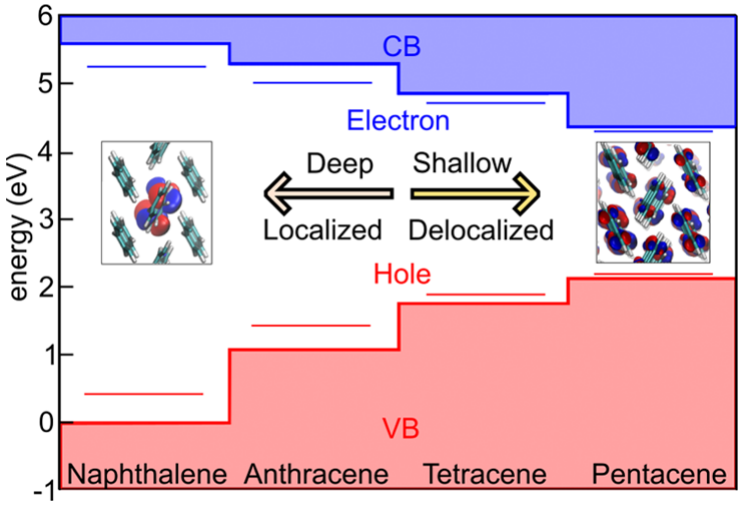

The performance of Koopmans-compliant hybrid functionals in reproducing the electronic structure of organic crystals is tested for a series of acene crystals. The calculated band gaps are found to be consistent with those achieved with the *GW* method at a fraction of the computational cost and in excellent accord with the experimental results at room temperature, when including the thermal renormalization. The energetics of excess holes and electrons reveals a struggle between polaronic localization and band-like delocalization. The consequences of these results on the transport properties of acene crystals are discussed.

The pioneering studies of the last century on the optoelectronic properties of organic semiconductors based on π-conjugated systems^1−4^ have paved the way for organics electronics.^5−7^ These materials have come under the spotlight of the scientific community for their favorable properties, in particular a good flexibility,^8−11^ making them attractive for a wide range of applications, including light-emitting diodes, thin-film transistors, and photovoltaics.^12−18^ In this context, the manifold routes of organic synthesis allow for a vast chemical and structural versatility of organic semiconductors. This should translate, in principle, into an immense variety of electronic properties to be tuned for the desired application.^19^ In fact, large sets of data, collected in the last decades, are available through online databases.^20,21^ However, a clear-cut structure–property correlation is still missing, thus hindering the development of organic semiconductors with optimal features. In particular, achieving a satisfactory charge carrier mobility via rational materials design is key for the technological implementation of organic semiconductors as light-absorbers or charge transport layers in third-generation photovoltaics.^22−26^

The charge transport mechanism in organic semiconductors is still under debate,^22,27,28^ since, as noted many times in the past, several theoretical methods have been able to predict results in good agreement with the experimental charge mobility,^23,29−33^ despite relying on different physical assumptions, ranging from hopping to band-like transport. However, it has been argued that both the hopping and the band-like mechanism may be inadequate for treating charge transport in organic semiconductors.^22,34−40^ Indeed, a genuine band-like mechanism is ruled out by the short mean free path observed in organic field-effect transistors,^27^ while hopping, usually used in conjunction with rate constants obtained by Marcus theory, predicts a thermally activated mechanism not fully consistent with experimental observations.^41^ That point of view has been questioned several times; indeed, the above observation is closely related to the use of classical theory of electron transfer. It has been demonstrated that the inclusion of quantum effects, nowadays extended to the whole bath provided by the intramolecular modes of redox units,^42^ allows obtaining a dependence of the mobilities on the temperature in fairly good agreement with the experimental data.^30,43−45^ Alternative models have been proposed, among which the transient localization theory is gaining an increasing popularity. This model is based on the idea that the unavoidable disorder in real crystals leads to a “transient localization” which would severely slow down carrier mobility. Nevertheless, time fluctuations of crystal disorder may still activate charge diffusion.^27,46^

In this context, reliable and affordable computational tools to determine the electronic properties of organic semiconductors can be game-changers, since the precise description of charge localization is paramount to define the proper charge transport mechanism.^22,27,30,32^ Simplified computational schemes, which avoid a full atomistic description of organic crystals by performing calculations on single molecules embedded in an electrostatic cavity,^47^ even if potentially useful for fast screening, cannot be employed to quantitatively describe other physical observables.^48^ Therefore, solid-state *ab initio* calculations remain the most obvious choice to assess the electronic properties of organic semiconductors. However, on one side, the use of highly accurate methods based on many-body perturbation theory (e.g., the *GW* method^49−52^), although possibly being appropriate for a benchmark on a few model systems,^53−56^ might be unaffordable for screening a plethora of materials. On the other side, density functional theory (DFT), the workhorse of computational materials science, fails in delivering quantitatively valid results,^57^ in terms of band gaps and charge localization/polaron binding energies, a consequence of the self-interaction error.^58,59^ While hybrid DFT methods^60−63^ generally lead to improved results, the accuracy of the calculated electronic structure is still partially undermined by self-interaction,^64^ as the amount of incorporated Fock exchange α is not satisfactorily defined. Since the band edges change linearly with α, while the position of the energy levels associated with localized electronic states is essentially stable, if referred to the average electrostatic potential of the system,^65^ errors in the opposite direction are possible, i.e., excessive localization and overestimation of polaron binding energies.

The recent idea of fixing the parameters of the density functional by imposing properties of the exact functional have ignited the development of nonempirical hybrid functionals.^66−72^ In exact DFT, the energy level of a single-particle state is independent from electron occupation (generalized Koopmans’ condition), thus ensuring the piecewise linearity of the functional upon fractional electron occupation.^73−75^ The definition of the embodied α via fulfillment of the Koopmans’ condition has given rise to the so-called Koopmans-compliant hybrid functionals, which were found to accurately reproduce the electronic properties (e.g., band gap, band edges, polaron binding energies) of both inorganic semiconductors and liquids at only a tiny fraction of the huge computational cost associated with the most advanced *GW* methods.^70,71,76−80^ Therefore, this class of hybrid functionals might represent the much-needed computational tool to explore the boundless chemical and structural *mare magnum* of organic semiconductors, provided that their accuracy is adequately tested and benchmarked.

To this end, acene crystals are convenient model objects, being the archetypal organic molecular crystals and thus subject to a large number of studies.^13^ In acene crystals, the building blocks, i.e., linear polycyclic aromatic hydrocarbons, are usually arranged in a herringbone structure (cf. Figure 1), with stabilization of the condensed phase provided by weak van der Waals interactions between molecules. A remarkably high degree of purity can be achieved for these crystals, which allows for in-depth experimental characterization of their intrinsic electronic properties.^81^

**Figure 1:**
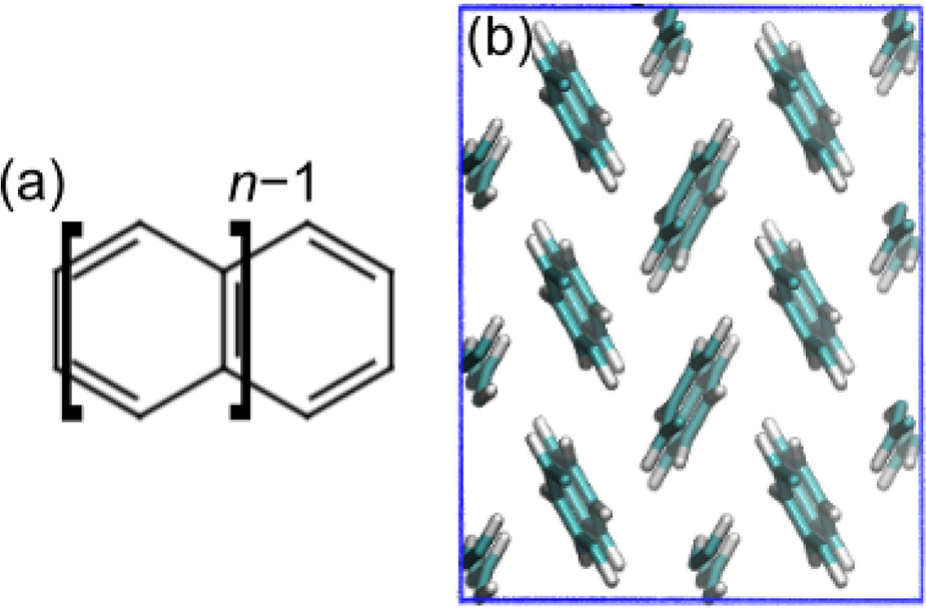
(a) General formula of linear acenes and (b) structural representation of the typical herringbone structure in which acene molecules crystallize, as observed from the 2 × 3 × 2 supercell employed for solid naphthalene. The *b* axis lies vertically.

In this study, we report on the performance of Koopmans-compliant functionals for the calculation of the electronic structure of the most common acene crystals, namely, naphthalene, anthracene, tetracene, and pentacene (cf. Figure 1). For each material, we consider the simplest hybrid-DFT scheme, based on the PBE0^60,61^ family of functionals, which was found to be provide results in line with those attained via Coulomb attenuated methods.^71,78,79^ We employ the probe method^71,78,79^ to determine the fraction of Fock exchange fulfilling the generalized Koopmans’ condition. To assess the performance of the Koopmans-compliant hybrid functionals, we benchmark them against results obtained using the *GW* approximation within the many-body perturbation theory. We perform one-shot *GW* calculations on top of energies and wave functions calculated within the Koopmans-compliant hybrid functionals. We include vertex corrections in the form of the bootstrap exchange–correlation kernel.^52,82^ This results in the so-called @PBE0(α_K_) scheme, which is expected to give accuracy close to that of the fully self-consistent *QSGW* method.^52,83^ We compare the results calculated at the hybrid and @PBE0(α_K_) levels of theory, and we find that both methods produce fundamental band gaps in very good accord with the experimental estimate at room temperature, provided that gap renormalization induced by thermal motion is properly included. We adopt the Koopmans-compliant functional to investigate charge localization in acene crystals, and from the calculated values of reorganization energies, we observe a struggle between polaronic localization and band-like delocalization, with the former being favored in naphthalene and anthracene and the latter more stable in tetracene and pentacene. Finally, we discuss how the present results can be related with charge transport properties observed for acene crystals.

We adopt the crystallographic structures obtained at room temperature,^84−87^ which is necessary because acene crystals show both a sizable gap increase upon thermal lattice expansion and renormalization due to room-temperature disorder (e.g., refs (53 and 56)). Space group, lattice parameters, and angles are reported in Table S1. To estimate the fraction of Fock exchange to be incorporated for the fulfillment of the generalized Koopmans’ condition, α_K_, we construct 2 × 3 × 2 supercells containing up to 864 atoms (cf. Figure 1 for naphthalene and the Supporting Information). Then, we insert a single hydrogen atom, thus introducing a localized state in the band gap of the organic semiconductor, e.g., crystalline anthracene in Figure 2. Then, we calculate the single-particle energy level for both the occupied (neutral supercell, H^0^) and unoccupied (positively charged, H^+^) states at the hybrid-DFT level, considering three different values of α. To alleviate the sizable computational cost associated with hybrid DFT for large supercells, we perform these calculations with the auxiliary density matrix method (ADMM) as implemented in the CP2K suite of programs (cf. the Supporting Information for computational details).^88−91^ The intersection between the linear evolution of the energy levels for occupied and empty states^71^ corresponds to α_K_ (cf. Figure 2). For comparison, we have repeated the procedure substituting H with F: the convergence of the −1/0 energy levels illustrated in Figure 2 evidences negligible differences in the determination of α_K_, confirming the robustness of the employed methodology. Calculated values of α_K_ (cf. Table 2) are found in a minimal range, from 0.37 (naphthalene) to 0.33 (pentacene), a result which can be interpreted in terms of the similar dielectric response of these materials.^92−94^

**Figure 2:**
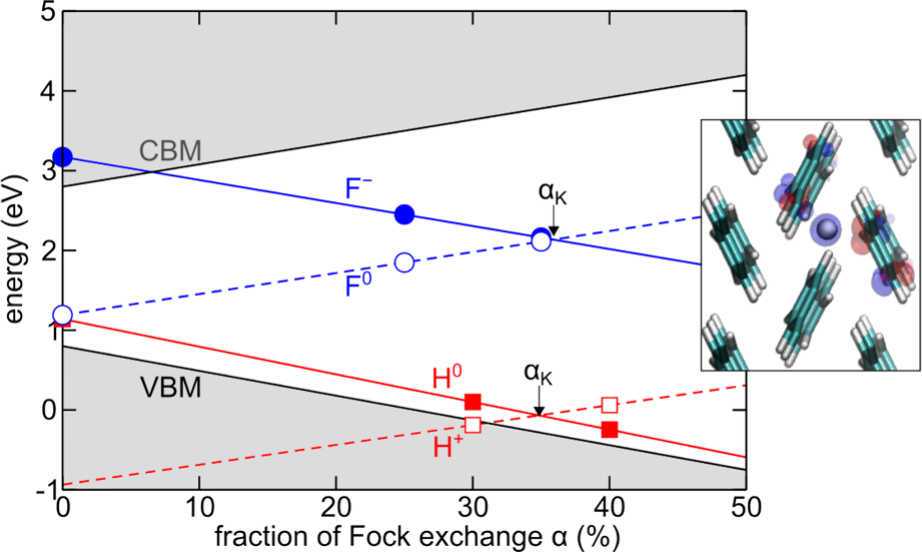
Occupied (full circles) and unoccupied (empty circles) single-particle energy levels of the interstitial hydrogen (red) and fluorine (blue) as a function of the fraction of Fock exchange α used in the PBE0 functional for solid anthracene. The α-dependent evolution of the valence band maximum (VBM) and the conduction band minimum (CBM) are also reported (solid black lines). Energies are referred to the average electrostatic potential. The intersection points, corresponding to α_K_, are highlighted. Inset: Licorice representation of the hydrogen probe (gray, enlarged) inserted in a periodic supercell of crystalline anthracene along with the isodensity representation of the corresponding highest occupied molecular orbital.

Inclusion of disorder and atomic vibrations is crucial to achieve a meaningful comparison with measurements performed at room temperature. Therefore, we evaluate the band gap renormalization, which is known to be relevant for small-molecule acene crystals.^56,95^ To this end, we perform Born–Oppenheimer molecular dynamics (MD) simulations at 300 K, in line with previous studies (cf. the Supporting Information for details),^96,97^ employing the rVV10 functional^98,99^ that self-consistently includes nonlocal electron correlation, which is required to accurately describe the structural features of acene crystals.^100−103^ From cell optimizations, we have verified that this functional produces lattice parameters in excellent agreement with those of the available low-temperature crystallographic data (cf. the Supporting Information), thus ensuring that the *NVT*-MD electronic structure is not affected by unphysical dynamics.

The thermal band gap renormalization Δ*E*_g_(*T*) is calculated at the PBE0(α_K_) level from 100 structural configurations equally spaced in time. In particular, we consider
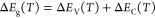
1where Δ*E*_V_(*T*) and Δ*E*_C_(*T*) are the individual contributions from the valence and conduction band edge, respectively, which are defined as
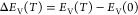
2and
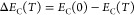
3where *E*_V_(*T*) and *E*_C_(*T*) are the valence and conduction band edges at room temperature, while *E*_V_(0) and *E*_C_(0) are those calculated on the ordered crystallographic structure. We extract these quantities through linear extrapolations of the wings of the electronic density of states (DOS) near the band edges in order to eliminate the effect of the band tail^83,104^ (cf. the Supporting Information).

Calculated values of Δ*E*_g_(*T*) collected in Table 1 show that the temperature-dependent renormalization is more pronounced for smaller acenes, namely, naphthalene and anthracene, a feature that has been recently explained in terms of a stronger coupling with the low-frequency phonons for these materials.^95^ The present results are consistent with those reported in the literature and achieved with electron–phonon self-energy,^56^ tight-binding models,^46^ and MD simulations on similar supercells.^95^

**Table 1:** Calculated Absolute Values of the Thermal Renormalization of the Valence and Conduction Band Edges, Δ*E*_V_(*T*) and Δ*E*_C_(*T*), Respectively, and of the Band Gap Δ*E*_g_(*T*), as Achieved from DFT-Based MD Simulations and Classical Molecular Mechanics (in parentheses)

material	Δ*E*_V_(*T*)	Δ*E*_C_(*T*)	Δ*E*_g_(*T*)
naphthalene	0.15	0.18	0.33 (0.30)
anthracene	0.10	0.15	0.25 (0.21)
tetracene	0.04	0.10	0.14 (0.11)
pentacene	0.03	0.05	0.12 (0.08)

Since DFT-based MD simulations are particularly expensive for the supercells that are necessary to adequately simulate acene crystals, we also consider, for comparison, the performance of classical molecular mechanics. To this end, we have constructed specific force fields for each acene (cf. the Supporting Information) and calculated Δ*E*_g_(*T*) adopting the same procedure. The gap renormalization (in parentheses in Table 1) is in fair agreement with the DFT-MD value with differences up to 0.04 eV for anthracene, thus suggesting that such a cheaper procedure may be safely employed in high-throughput computational protocols.

We now focus on the fundamental band gaps of a set of acene crystals using the @PBE0(α_K_) scheme at 0 K, *E*_g_(0). The results are presented in Table 2. We observe that the band gaps obtained at the @PBE0(α_K_) level are in good agreement with those calculated using the Koopmans-compliant hybrid functional, indicating that our one-shot procedure is reasonably close to self-consistency. This also confirms the effectiveness of Koopmans-compliant hybrid functionals in this class of materials. Furthermore, we compare our results with previous *GW* calculations by Rangel et al.,^56^ who employed both *G*_0_*W*_0_ and ev*GW* methods for a similar set of materials. The ev*GW* method partially achieves self-consistency with respect to the eigenvalues. Our calculated band gaps are generally smaller than those obtained using ev*GW* in ref (56), which is likely due to the inclusion of vertex corrections in our approach.

**Table 2:** Calculated Values of α_K_ (%), at 0 K and Room Temperature (RT), Band Gaps (in eV) at the PBE0(α_K_) and *G*_0_*W̃*_0_@PBE0(α_K_) Levels of Theory along with the Experimental Estimates^a^

material	α_K_	PBE0(α_K_) 0 K	*G*_0_*W̃*_0_@PBE0(α_K_) 0 K	PBE0(α_K_) RT	*G*_0_*W̃*_0_@PBE0(α_K_) RT	exptl
naphthalene	37	5.56 (5.58)	5.68	5.23 (5.25)	5.35	5.0–5.4^105,106^
anthracene	35	4.12 (4.20)	4.26	3.86 (3.95)	4.01	3.9–4.0^105,107^
tetracene	34	2.86 (3.04)	2.97	2.76 (2.90)	2.83	2.8–3.1^107,108^
pentacene	33	2.26 (2.22)	2.25	2.14 (2.10)	2.13	2.1–2.2^109,110^

a In parentheses, the values calculated with the ADMM/supercell approach are reported. Δ*E*_g_(*T*) as calculated from DFT-MD molecular dynamics is included in the RT values.

Next, we evaluate the accuracy of our computational scheme in predicting the band gaps of acenes at room temperature by comparing with experimental data. To do so, we define the theoretical room-temperature band gap as *E*_g_(*T*) = *E*_g_(0) – Δ*E*_g_(*T*). As shown in Table 2, our predictions of band gaps at room temperature based on @PBE0(α_K_) calculations are in good agreement with the experimental values for all materials. For comparison, we also present the band gaps calculated at the PBE0(α_K_) level using the ADMM/supercell computational scheme employed to determine α_K_. The consistent agreement between our predictions and the experimental data indicates that this method can be reliably applied to study the electronic structure of organic materials.

We now investigate charge localization in the acene family. To this end, we perform structural relaxation of the supercells with an extra hole and an electron. This allows us to define the reorganization energy of the acene crystal
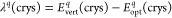
4as the difference between the total energy of the relaxed charged supercell  and that with the nuclei fixed to the equilibrium positions of the neutral system , the latter representing the energy associated with charge delocalization. We note that the positive value of λ indicates that localization is favorable, while the negative value suggests that the localized state is not stable. Furthermore, to disentangle the different effects contributing to the stabilization of the excess charges, we adopt the following computational protocol: First, for each acene, we isolate a single molecule from the supercell, we place it in vacuum, and we perform a structural relaxation with an excess charge *q*. This allows localizing *q* on a single molecule and defining the molecular reorganization energy
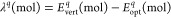
5as the total-energy difference between the isolated molecule upon vertical injection of the charge  and the optimized molecule . Then, the charged molecule is reintroduced in the supercell, thus replacing that previously extracted. This model allows us to calculate the energy gain associated with localization of the charge on a single molecule λ^*q*^(loc) with respect to delocalization on the band edge:
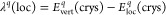
6where *E*_loc_^*q*^(crys) is the total energy of the supercell with *q* localized on a single molecule. Since we have verified that full relaxation of the charged supercells starting either from the structure of the neutral crystal or from the localized model produces the same final structure, the effect of the environment λ^*q*^(env) is evaluated as
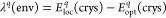
7from which it follows that
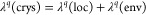
8For a visual aid, the different reorganization energies are summarized in Scheme 1.

**Scheme 1:**
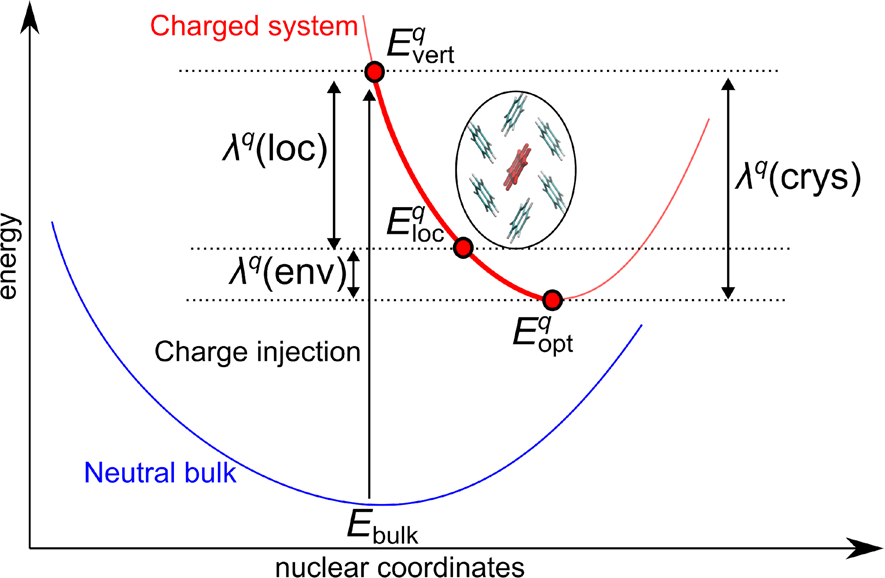
Schematic Representation of the Calculated Reorganization Energies, as Defined in the Main Text The scheme refers to the cases for which charge localization on a single molecule is more stable than the system upon vertical charge injection.

Reorganization energies associated with hole and electron injection, λ^+^ and λ^–^, respectively, are collected in Table 3. First, we note that the present results on the single molecules are in line with previous calculations and experiments, with the observed trends of decreasing λ^*q*^(mol) for longer acenes and slightly larger values of reorganization for electrons nicely reproduced.^111−113^

**Table 3:** Calculated Values of Reorganization Energies (cf. main text for details) for the Acene Crystals Considered in This Study^a^

	hole localization			
	λ^+^(mol)	λ^+^(loc)	λ^+^(env)	λ^+^(crys)
naphthalene	0.11	0.14	0.08	0.22
anthracene	0.09	0.14	0.06	0.20
tetracene	0.08	–0.04	0.09^b^	0.05
pentacene	0.06	–0.08	0.10^b^	0.02

	electron localization			
	λ^–^(mol)	λ^–^(loc)	λ^–^(env)	λ^–^(crys)
naphthalene	0.15	0.15	0.12	0.27
anthracene	0.12	0.10	0.08	0.18
tetracene	0.10	–0.05	0.11^b^	0.06
pentacene	0.08	–0.07	0.08^b^	0.01

a All values are given in eV.

b Values do not refer to an additional stabilization of localized charges given by the environment as in these cases charge localization is unfavorable.

Data calculated on supercells allow distinguishing two different behaviors upon charge injection, with minor differences between holes and electrons. In fact, for naphthalene and anthracene crystals, values of λ^*q*^(loc) above 0.1 eV indicate that polaronic localization of the charge on a single molecule is favorable with respect to delocalization on the band edge states. Furthermore, the response of the surrounding molecules denoted by λ^*q*^(env) enhances the stabilization of the charge. The latter remains largely confined on one molecule, whose nuclei positions almost do not change during relaxation, thus indicating an exiguous charge transfer toward surrounding molecules (cf. Figure 3), in line with a very recent study showing that polaronic localization in naphthalene arises from intramolecular phonons.^114^ We further note that the calculated λ^*q*^(env) values are somewhat larger than those from previous study in which this contribution however was evaluated from classical molecular mechanics.^115^ Nevertheless, the trends are qualitatively consistent.

**Figure 3:**
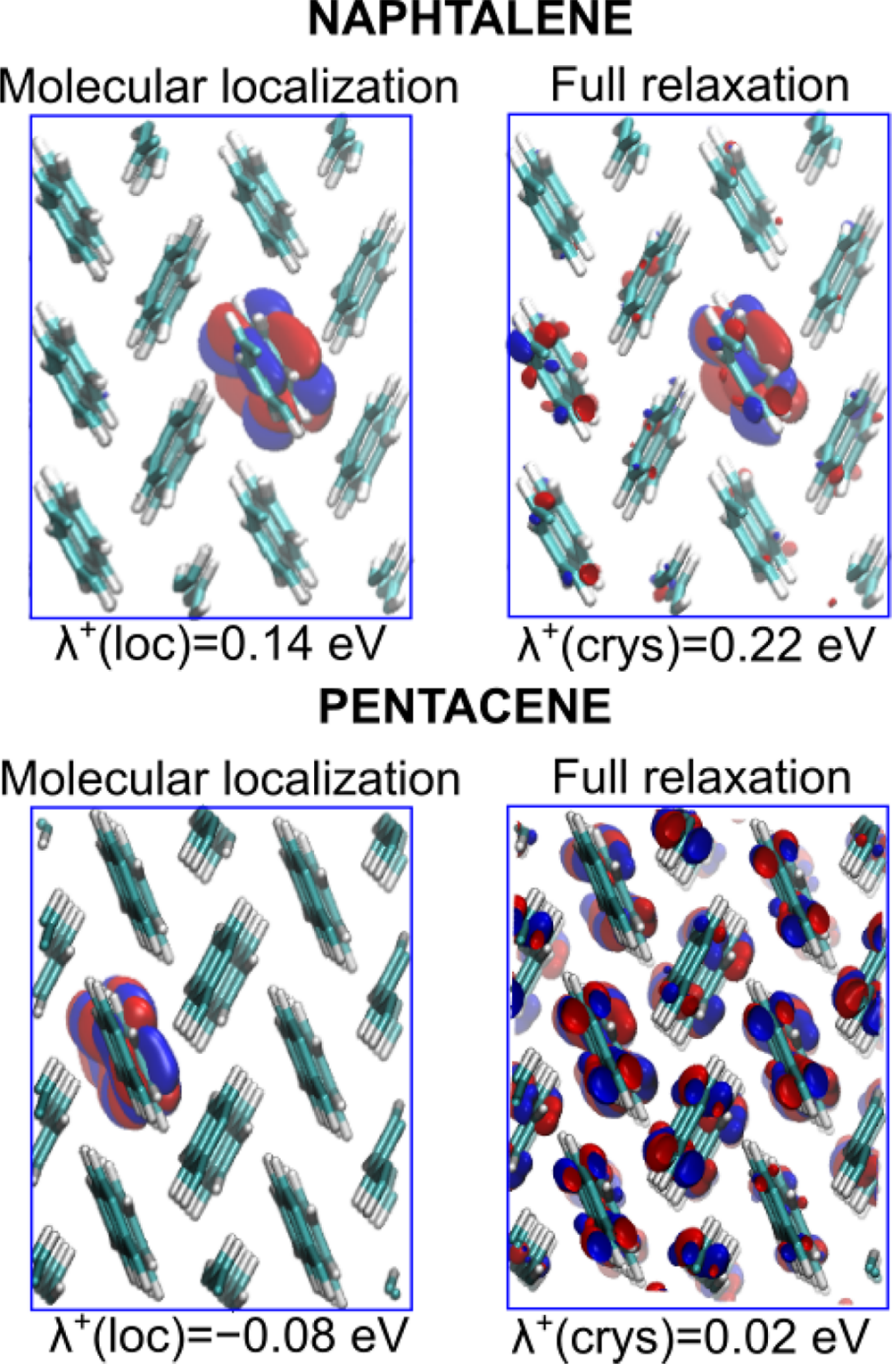
Isodensity representation of the lowest unoccupied molecular orbital (LUMO) for positively charged supercells of crystalline naphthalene (top) and pentacene (bottom). Left panels depict charge localization on a single molecule (cf. main text), while right panels illustrate the LUMO of the fully relaxed charged supercell.

In stark contrast, the negative values of λ^*q*^(loc) for tetracene and pentacene imply that charge localization is slightly unfavorable in these cases; that is, the localized states are essentially resonant with band edges. In fact, upon full relaxation of the charged system, both holes and electrons are found to be substantially delocalized, as evidenced by the extremely small values of λ^*q*^(crys) and by the analysis of the pertinent molecular orbitals (cf. pentacene in Figure 3). This is accompanied mainly by relaxation of the molecule bearing the charge which restores its structural configuration in the neutral crystal.

Finally, we calculate the charge transition levels of the polaronic defects with respect to the band edges of each acene, using a grand-canonical formulation of defects in crystalline materials.^116,117^ Within this theory, the formation energy of a defect *X* in a charge state *q* is
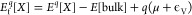
9where *E*[bulk] and *E*^*q*^[*X*] are the total energies of the pristine bulk of the supercell bearing the defect, μ is the electron chemical potential, and ϵ_V_ is the valence band edge of the semiconductor. The energy level corresponding to a transition from a charge state *q* to *q*′ of a defect *X* is defined as the Fermi level for which their formation energies are equal, :
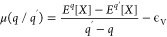
10For polarons, *E*^*q*^[*X*] = *E*^+^[loc], *E*^–^[loc] for localized hole and electron respectively, while .^77,118^ Therefore
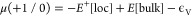
11and
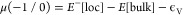
12

In Figure 4, the polaronic charge transition levels, as calculated from eqs 11 and 12, are reported for each acene crystal. The energy levels of different acenes are aligned via the measured valence band spectra of ref (119), and the hybrid-DFT 0 K values of the band gaps from Table 2 are considered. In line with the observed values of λ, calculated energy levels for localized holes (electrons) in naphthalene and anthracene are clearly above (below) the valence (conduction) band edge. At variance with this, tetracene and pentacene feature shallow energy levels, essentially resonant with the respective band edges. We note that, while thermal renormalization reduces the band gap, thus possibly affecting the energy diagram, at the same time, room-temperature disorder is known to stabilize localized charges in both organic and inorganic semiconductors^77,120,121^ and will deserve further consideration.

**Figure 4:**
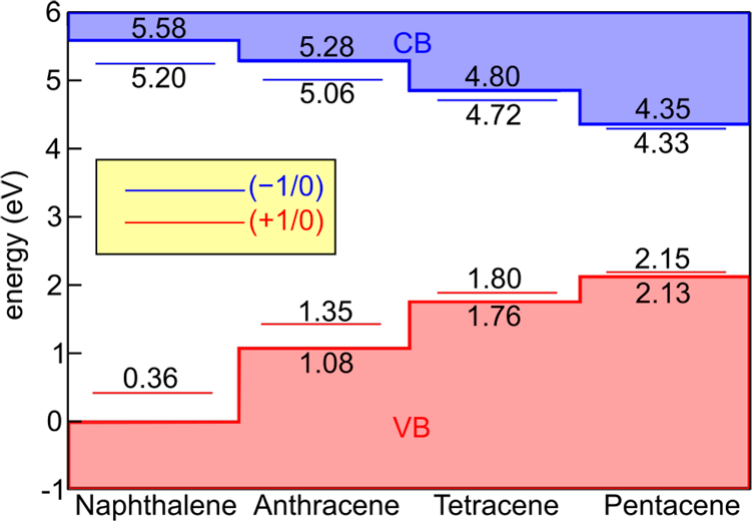
Calculated polaronic charge transition levels (cf. eqs 11 and 12, main text) for the considered acene crystals. Energies are referred to the valence band of naphthalene and are aligned via the valence band spectra of ref (119). For consistency, 0 K band gaps from Table 2 are considered.

The struggle between localization and delocalization denoted by the calculated values of λ and of the charge transition levels can help rationalizing the transport properties of these materials, which have long puzzled the research community as they typically fall in a transport regime at the boundaries between the limit cases describable with either band transport or charge hopping.^29,120,122^ In this context, our computational analysis suggests that a small or midsized polaron hopping model might be justified for naphthalene and anthracene. However, such a model cannot hold for tetracene and pentacene as our analysis shows larger delocalization of charge carriers. Therefore, band-like/large polaron models are preferable, in line with the view recently proposed on the basis of nonadiabatic molecular dynamics.^25,32^

In conclusion, we have shown that Koopmans-compliant hybrid functionals represent a reliable and efficient tool to rapidly evaluate the band gap of acene crystals, with results consistent with those achieved with the *GW* method and in excellent agreement with experimental results, when thermal renormalization of the gap is properly accounted for. The employed methodology, devoid of the self-interaction error, allowed for an in-depth analysis of charge localization in different acenes, which highlighted a struggle between localization and delocalization of the charge carriers: for shorter acenes, namely naphthalene and anthracene, polaronic localization appears to be energetically favored; at variance with this, holes and electrons in tetracene and pentacene are endowed with a larger delocalization. Overall, the present results may aid the rationalization of the charge transport properties of acene crystals and the use of the presented methodology can be extended to other organic crystals.

## Supporting Information Available

Computational details, benchmark of the employed rVV10 functional on low-temperature crystallographic structures, linear extrapolation of the electronic density of states for the calculation of the band gap, and additional references (PDF)
