# Supplementary material for: Charge Localization in Acene Crystals from Ab Initio Electronic Structure
Source: J Phys Chem Lett. 2023 Mar 30;14(13):3343–51. doi: 10.1021/acs.jpclett.3c00191 (PMC10084468; doi:10.1021/acs.jpclett.3c00191)
Supplement: Supplementary file 1 — jz3c00191_si_001.pdf [file jz3c00191_si_001.pdf]

Supplementary Information for: Charge Localization in Acene Crystals from *Ab Initio* Electronic Structure

Francesco Ambrosio,[*,†,‡] Julia Wiktor,[*,¶] Alessandro Landi,[†] and Andrea Peluso[†]

†*Dipartimento di Chimica e Biologia Adolfo Zambelli, Università di Salerno, Via Giovanni Paolo II, I-84084 Fisciano (SA), Italy*

‡*Dipartimento di Scienze, Università degli Studi della Basilicata, Viale dell'Ateneo Lucano, 10 - 85100 Potenza, Italy*

¶*Department of Physics, Chalmers University of Technology, SE-412 96 Gothenburg, Sweden*

E-mail: fambrosio@unisa.it; julia.wiktor@chalmers.se

Computational Details

The $2 \times 3 \times 2$ supercells (cf. Figure S1) employed to determine α_K are constructed starting from the crystallographic structures solved at room temperature,[1–4] for which space group, lattice parameters, and angles are reported in Table S1. Each supercell contains 24 acene molecules.

Table S1: Experimental lattice parameters a, b, c (Å) and angles α, β, γ (degrees) of the room-temperature unit cells for the acene crystals studied in this work[1–4] as available from the Cambridge Structural Database (CSD)[5]

	Space group	a	b	c	α	β	γ
Naphthalene	$P2_1\backslash a$	8.26	5.98	8.67	90	122.73	90
Anthracene	$P2_1\backslash a$	8.55	6.02	11.17	103.51	90	90
Tetracene	$P\bar{1}$	7.98	6.14	13.57	101.35	100.44	92.50
Pentacene	P1	7.93	6.14	16.03	101.90	112.60	85.80

Electronic structure calculations on supercells are performed using the freely available CP2K \QUICKSTEP package,[6] which combines atomic basis sets with an auxiliary plane-wave basis set to re-expand the electron density. In particular, we use the MOLOPT double-zeta polarized basis set and a cutoff of 800 Ry for the plane waves. Core electrons are treated with the analytical Goedecker-Teter-Hutter pseudopotentials.[7] Furthermore, we employ the auxiliary density matrix method, as implemented in CP2K, with the cFIT auxiliary basis set,[8,9] to speed up the calculation of exchange integrals in hybrid functional calculations.[8,9] For the determination of α_K to be introduced in the PBE0(α) functional, we note that the energies of the Kohn-Sham levels of localized state in charged supercells are subject to an electrostatic finite-size error, arising from periodic boundary conditions.[10] Therefore, we employ the correction term derived in Ref. 11 for Kohn-Sham levels, which is connected with the Freysoldt-Neugebauer-Van de Walle (FNV) scheme commonly employed for total energies.[10,12] Corrections for each employed supercell are reported in Table S2. These terms are calculated considering the experimental high-frequency dielectric constant of each acene crystal,[13–15] as computations are performed without relaxing the positions of the nuclei.

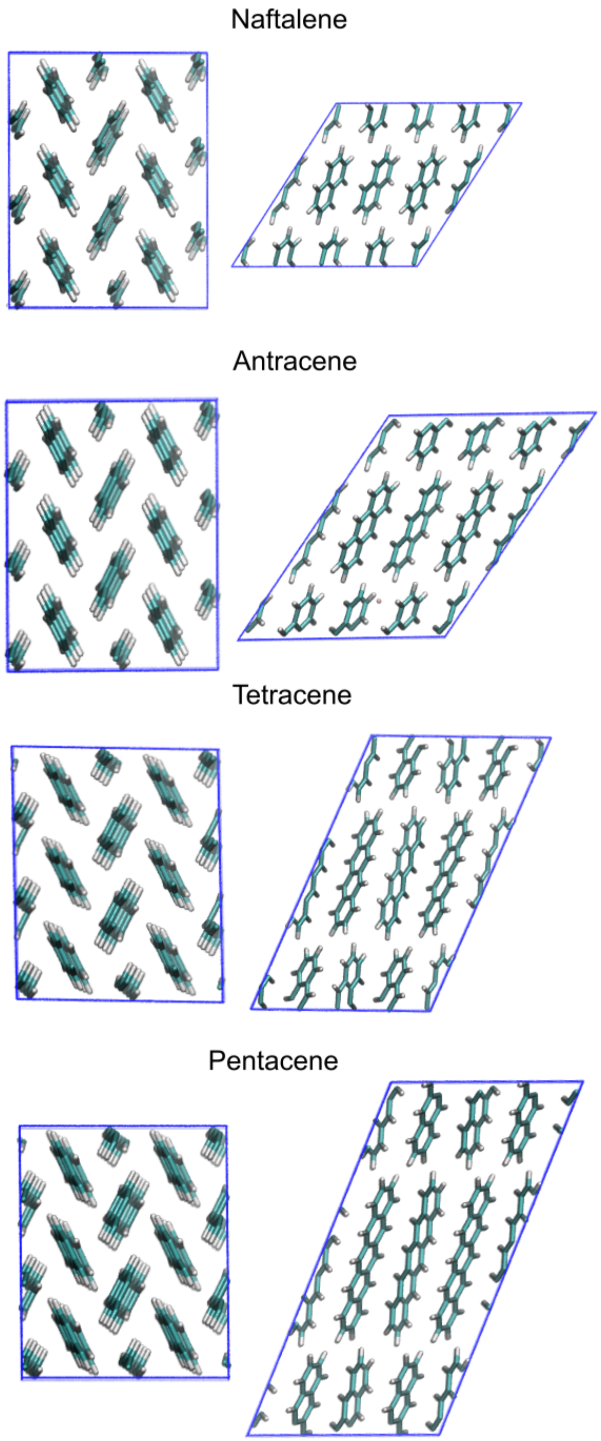

Figure S1: Licorice representation of the periodic supercells employed in this work to model the room-temperature crystalline structures of (a) naphthalene, (b) anthracene, (c) tetracene, and (d) pentacene. C atoms in cyan, H in white. In left panels, the b axis lies vertically, while in right panels is perpendicular to the plane.

Table S2: Absolute value of the electrostatic finite-site correction on the Kohn-Sham energy level associated with the localized state induced by the probe in charged supercells. Values are given in eV.

Material	Correction
Naphthalene	1.00
Anthracene	0.90
Tetracene	0.56
Pentacene	0.54

Band gap calculations using the *GW* approximation within the many-body perturbation theory are carried out in the ABINIT code.[16,17] In these calculations, we use the eigenvalues and wavefunctions from the PBE0(α_K) calculation as a starting point and we include an efficient exchange–correlation kernel to account for vertex corrections.[18] Core–valence interactions are treated through norm-conserving pseudopotentials.[19,20] In all calculations, we include at least 500 unoccupied bands. The frequency dependence of the self-energy is accounted for through the plasmon-pole approximation.[21] For all materials, we set the ground-state cutoff energy to 42 Ry. We use the cutoff energy of 10 Ry for the representation of the wavefunction and the exchange part of the self-energy operator, and a cutoff energy of 5 Ry to represent the independent-particle susceptibility. The calculations are carried out in unit cells containing between 36 and 72 atoms and the **k**-point sampling is set to 4×4×4. We verified on the case of naphthalene that these parameters lead to band gaps converged within 0.05eV.

DFT-based Born-Oppenheimer MD simulations in the NVT ensemble are carried out in periodic supercells, as previously described. The target temperature, controlled by a Nosé-Hoover thermostat,[22,23] is set to 300 K and a time-step of 0.50 fs is employed. Production runs of 5 ps are preceded by an equilibration run of 5 ps. MD simulations are performed employing the rVV10 functional,[24,25] which includes self-consistently non-local electron correlation and it is known to correctly describe the energetics of $\pi - \pi$ interactions[26]. Furthermore, it is here found to ensure a reliable description of the structural properties of low-temperature

acene crystals (*vide infra*).

In fact, to evaluate the performance of the employed rVV10 functional, we consider, as reference, the crystallographic structures achieved from low-temperature X-ray diffraction experiments,[2,27–29] retrieved from the CSD[5]. Therefore, we construct $2 \times 3 \times 2$ supercells for naphthalene and anthracene and $3 \times 2 \times 2$ supercells for tetracene and pentacene. Then, we perform, for each system, a cell optimization in which the lattice parameters are allowed to relax, and we compare the calculated values with the experimental ones. Data collected in Table S3 denote minimal difference between calculated and measured values, with a maximum error (for tetracene) still below 2%.

Table S3: Experimental[2,27–29] and calculated lattice parameters a, b, c of $2\times3\times2$ (naphthalene and anthracene) and $3\times2\times2$ (tetracene and pentacene) supercells of the low-temperature X-ray crystal structures of the studied acenes. All values are given in Å.

	low-temperature X-ray			rVV10		
	a	b	c	a	b	c
Naphthalene	16.216	17.819	17.294	15.916	17.614	17.118
Anthracene	16.829	17.936	22.246	16.676	17.689	22.018
Tetracene	18.169	15.675	26.021	17.872	15.353	25.884
Pentacene	18.717	15.272	28.660	18.536	15.153	28.715

In order to perform classical molecular mechanics (MM) simulations, we construct adequate force fields for naphtalene, anthracene, tetracene and pentacene. we first obtain equilibrium geometries, normal modes, and vibrational frequencies for these molecules in their neutral form at the DFT level using the B3LYP functional, as implemented in the Gaussian16 package,[30] including Grimme's D3 dispersion,[31] with the 6-31G(d) basis set, which has been proved to yield sufficiently accurate results.[32,33] Then, we use point charges and inter-molecular Lennard-Jones parameters from appropriate OPLS atom types[34], while the intra-molecular parameters (equilibrium positions and force constants) have been obtained by parametrizing a quantum-mechanically derived force fields (QMD-FFs), fitting QM reference data with the Joyce software.[35–38]

Classical MM simulation are carried out with the GROMACS2020.5 software[39] employing

$2\times3\times2$ supercell starting from the crystallographic data, as done for DFT-MD simulations. We adopt periodic boundary conditions and took into account long range electrostatic effects through the Particle-Mesh-Ewald algorithm.[40] We use an integration time step of 2 fs, imposing constraints on the bonds involving H atoms through the LINCS algorithm,[41] adopting a modified Berendsen thermostat[42] to control temperature and a Parrinello-Rahman barostat to control pressure.[43] The computational protocol consist of an initial steepest descent minimisation, followed by an initial NVT equilibration in at 300 K over 100 ps. Finally, we carry out a production run of 1 ns in the NVT ensemble, in order to keep the experimental cell parameters.

All the relevant input files of first-principles molecular dynamics, hybrid-functional and *GW* calculations, as well as the structural configurations employed to calculate the band gap renormalization are available at: `https://github.com/XelaleX1/Paper_acenes_JPCL`

Thermal renormalization of the band gap

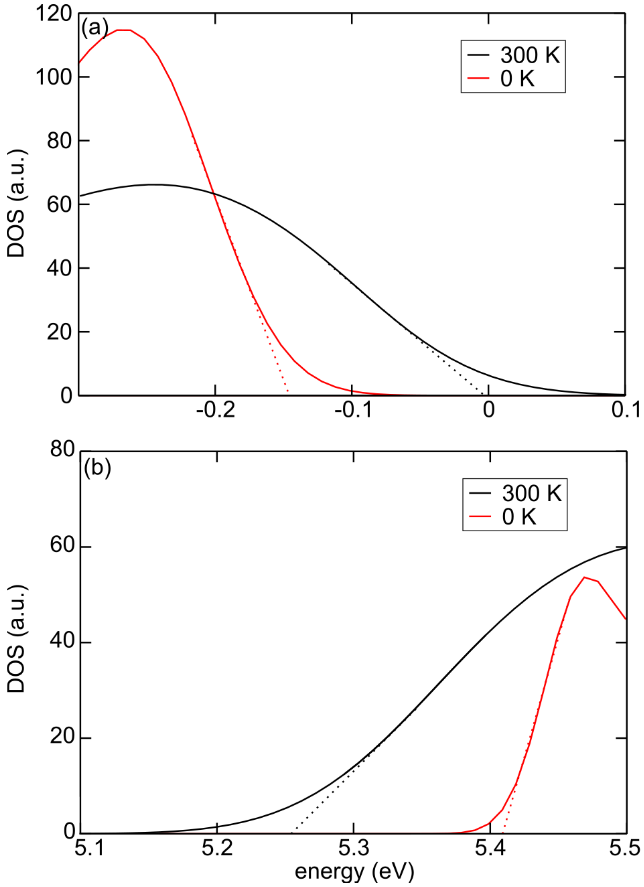

Figure S2: Comparison of the density of states (DOS) of crystalline naphthalene calculated using the PBE0(α_{K} functional at 0 K and taking averages over the MD trajectory at 300 K. We use a broadening of 0.05 eV to generate the plots. PAnels (a) and (b) show the valence and conduction edges, respectively. Dashed lines indicate the linear extrapolations used to determine the positions of the valence and conduction band edges. Energies are aligned with respect to the C 2s core level and referred with respect to the linearly extrapolated valence band edge at 300 K.

References

(1) Capelli, S. C.; Albinati, A.; Mason, S. A.; Willis, B. T. Molecular Motion in Crystalline Naphthalene: Analysis of Multi-Temperature X-ray and Neutron Diffraction Data. *J. Phys. Chem. A* **2006**, *110*, 11695–11703.

(2) Brock, C. P.; Dunitz, J. Temperature Dependence of Thermal Motion in Crystalline Anthracene. *Acta Crystallogr. B: Struct. Sci. Cryst. Eng. Mater.* **1990**, *46*, 795–806.

(3) Robertson, J. M.; Sinclair, V.; Trotter, J. The Crystal and Molecular Structure of Tetracene. *Acta Crystallogr.* **1961**, *14*, 697–704.

(4) Campbell, R.; Robertson, J. M.; Trotter, J. The Crystal and Molecular Structure of Pentacene. *Acta crystallogr.* **1961**, *14*, 705–711.

(5) Groom, C. R.; Bruno, I. J.; Lightfoot, M. P.; Ward, S. C. The Cambridge Structural Database. *Acta Cryst. B* **2016**, *72*, 171–179.

(6) VandeVondele, J.; Krack, M.; Mohamed, F.; Parrinello, M.; Chassaing, T.; Hutter, J. Quickstep: Fast and Accurate Density Functional Calculations Using a Mixed Gaussian and Plane Waves Approach. *Comput. Phys. Commun.* **2005**, *167*, 103 – 128.

(7) Hartwigsen, C.; Goedecker, S.; Hutter, J. Relativistic Separable Dual-Space Gaussian Pseudopotentials from H to Rn. *Phys. Rev. B* **1998**, *58*, 3641–3662.

(8) Guidon, M.; Schiffmann, F.; Hutter, J.; VandeVondele, J. Ab Initio Molecular Dynamics Using Hybrid Density Functionals. *J. Chem. Phys.* **2008**, *128*, 214104.

(9) Guidon, M.; Hutter, J.; VandeVondele, J. Auxiliary Density Matrix Methods for Hartree-Fock Exchange Calculations. *J. Chem. Theory Comput.* **2010**, *6*, 2348–2364.

(10) Freysoldt, C.; Neugebauer, J.; Van de Walle, C. G. Fully *Ab Initio* Finite-Size Corrections for Charged-Defect Supercell Calculations. *Phys. Rev. Lett.* **2009**, *102*, 016402.

(11) Chen, W.; Pasquarello, A. Correspondence of Defect Energy Levels in Hybrid Density Functional Theory and Many-Body Perturbation Theory. *Phys. Rev. B* **2013**, *88*, 115104.

(12) Komsa, H.-P.; Rantala, T. T.; Pasquarello, A. Finite-Size Supercell Correction Schemes for Charged Defect Calculations. *Phys. Rev. B* **2012**, *86*, 045112.

(13) Ishii, K.; Kinoshita, M.; Kuroda, H. Dielectric Constant Measurement on Organic Crystalline Powder. *Bull. Chem. Soc. Jpn.* **1973**, *46*, 3385–3391.

(14) Del Carro, P.; Camposeo, A.; Persano, L.; Tavazzi, S.; Campione, M.; Papagni, A.; Raimondo, L.; Silvestri, L.; Spearman, P.; Cingolani, R., et al. Monolithic Vertical Microcavities Based on Tetracene Single Crystals. *Applied Physics Letters* **2008**, *92*, 41.

(15) Faltermeier, D.; Gompf, B.; Dressel, M.; Tripathi, A. K.; Pflaum, J. Optical Properties of Pentacene Thin Films and Single Crystals. *Phys. Rev. B* **2006**, *74*, 125416.

(16) Gonze, X.; Amadon, B.; Anglade, P.-M.; Beuken, J.-M.; Bottin, F.; Boulanger, P.; Bruneval, F.; Caliste, D.; Caracas, R.; Côté, M., et al. ABINIT: First-Principles Approach to Material and Nanosystem Properties. *Computer Physics Communications* **2009**, *180*, 2582–2615.

(17) Gonze, X.; Jollet, F.; Araujo, F. A.; Adams, D.; Amadon, B.; Applencourt, T.; Audouze, C.; Beuken, J.-M.; Bieder, J.; Bokhanchuk, A., et al. Recent Developments in the ABINIT Software Package. *Computer Physics Communications* **2016**, *205*, 106–131.

(18) Chen, W.; Pasquarello, A. Accurate Band Gaps of Extended Systems via Efficient Vertex Corrections in GW. *Physical Review B* **2015**, *92*, 041115.

(19) Hamann, D. Optimized Norm-Conserving Vanderbilt Pseudopotentials. *Physical Review B* **2013**, *88*, 085117.

(20) van Setten, M. J.; Giantomassi, M.; Bousquet, E.; Verstraete, M. J.; Hamann, D. R.; Gonze, X.; Rignanese, G.-M. The PseudoDojo: Training and Grading a 85 Element Optimized Norm-Conserving Pseudopotential Table. *Computer Physics Communications* **2018**, *226*, 39–54.

(21) Godby, R. W.; Needs, R. Metal-insulator transition in Kohn-Sham theory and quasiparticle theory. *Physical review letters* **1989**, *62*, 1169.

(22) Nosé, S. A unified Formulation of the Constant Temperature Molecular Dynamics Methods. *J. Chem. Phys.* **1984**, *81*, 511–519.

(23) Hoover, W. G. Canonical Dynamics: Equilibrium Phase-Space Distributions. *Phys. Rev. A* **1985**, *31*, 1695–1697.

(24) Vydrov, O. A.; Van Voorhis, T. Nonlocal van der Waals Density Functional: The Simpler the Better. *J. Chem. Phys.* **2010**, *133*, 244103.

(25) Sabatini, R.; Gorni, T.; de Gironcoli, S. Nonlocal van der Waals Density Functional Made Simple and Efficient. *Phys. Rev. B* **2013**, *87*, 041108.

(26) Mardirossian, N.; Ruiz Pestana, L.; Womack, J. C.; Skylaris, C.-K.; Head-Gordon, T.; Head-Gordon, M. Use of the rVV10 Nonlocal Correlation Functional in the B97M-V Density Functional: Defining B97M-rV and Related Functionals. *J. Phys. Chem. Lett.* **2017**, *8*, 35–40.

(27) Brock, C. P.; Dunitz, J. D. Temperature Dependence of Thermal Motion in Crystalline Naphthalene. *Acta Crystallogr. B Struct. Cryst. Cryst. Chem.* **1982**, *38*, 2218–2228.

(28) Holmes, D.; Kumaraswamy, S.; Matzger, A. J.; Vollhardt, K. P. C. On the Nature of Nonplanarity in the [N] Phenylenes. *Eur. J. Chem.* **1999**, *5*, 3399–3412.

(29) Mattheus, C. C.; Dros, A. B.; Baas, J.; Meetsma, A.; De Boer, J. L.; Palstra, T. T.

Polymorphism in Pentacene. *Acta Crystallogr. C Struct. Chem. Comm.* **2001**, *57*, 939–941.

(30) Frisch, M. J. et al. Gaussian 16 Revision C.01. 2016; Gaussian Inc. Wallingford CT.

(31) Grimme, S.; Antony, J.; Ehrlich, S.; Krieg, H. A Consistent and Accurate Ab Initio Parametrization of Density Functional Dispersion Correction (DFT-D) for the 94 Elements H-Pu. *J. Chem. Phys.* **2010**, *132*, 154104.

(32) Landi, A.; Troisi, A. Rapid Evaluation of Dynamic Electronic Disorder in Molecular Semiconductors. *J. Phys. Chem. C* **2018**, *122*, 18336–18345.

(33) Nguyen, T. P.; Shim, J. H.; Lee, J. Y. Density Functional Theory Studies of Hole Mobility in Picene and Pentacene Crystals. *J. Phys. Chem. C* **2015**, *119*, 11301–11310.

(34) Jorgensen, W. L.; Maxwell, D. S.; Tirado-Rives, J. Development and Testing of the OPLS All-Atom Force Field on Conformational Energetics and Properties of Organic Liquids. *J. Am. Chem. Soc.* **1996**, *118*, 11225–11236.

(35) Cacelli, I.; Prampolini, G. Parametrization and Validation of Intramolecular Force Fields Derived from DFT Calculations. *J. Chem. Theory Comput.* **2007**, *3*, 1803–1817.

(36) Barone, V.; Cacelli, I.; De Mitri, N.; Licari, D.; Monti, S.; Prampolini, G. Joyce and Ulysses: Integrated and User-Friendly Tools for the Parameterization of Intramolecular Force Fields from Quantum Mechanical Data. *Phys. Chem. Chem. Phys.* **2013**, *15*, 3736–51.

(37) Cerezo, J.; Prampolini, G.; Cacelli, I. Developing Accurate Intramolecular Force Fields for Conjugated Systems Through Explicit Coupling Terms. *Theor. Chem. Acc.* **2018**, *137*, 80.

(38) Landi, A.; Padula, D. Multiple Charge Separation Pathways in New-Generation Non-Fullerene Acceptors: a Computational Study. *J. Mater. Chem. A* **2021**, *9*, 24849–24856.

(39) Abraham, M. J.; Murtola, T.; Schulz, R.; Páll, S.; Smith, J. C.; Hess, B.; Lindahl, E. GROMACS: High Performance Molecular Simulations Through Multi-Level Parallelism from Laptops to Supercomputers. *SoftwareX* **2015**, *1-2*, 19–25.

(40) Darden, T.; York, D.; Pedersen, L. Particle mesh Ewald: AnN·log(N) Method for Ewald Sums in Large Systems. *J. Chem. Phys.* **1993**, *98*, 10089–10092.

(41) Hess, B.; Bekker, H.; Berendsen, H. J. C.; Fraaije, J. G. E. M. LINCS: A Linear Constraint Solver for Molecular Simulations. *J. Comput. Chem.* **1997**, *18*, 1463–1472.

(42) Berendsen, H. J. C.; Postma, J. P. M.; van Gunsteren, W. F.; DiNola, A.; Haak, J. R. Molecular Dynamics with Coupling to an External Bath. *J. Chem. Phys.* **1984**, *81*, 3684–3690.

(43) Parrinello, M.; Rahman, A. Polymorphic Transitions in Single Crystals: A New Molecular Dynamics Method. *J. Appl. Phys.* **1981**, *52*, 7182–7190.
